# Supplementary material for: Mid-zone hepatocytes trade proliferation for survival via Atf4-Chop axis in early acute liver injury
Source: eLife. 2026 Oct 26;14:RP108646. doi: 10.7554/eLife.108646 (PMC13707374; doi:10.7554/eLife.108646)
Supplement: Supplementary file 1 [file elife-108646-fig6-figsupp1-data2.pdf]

Raw unedited membranes

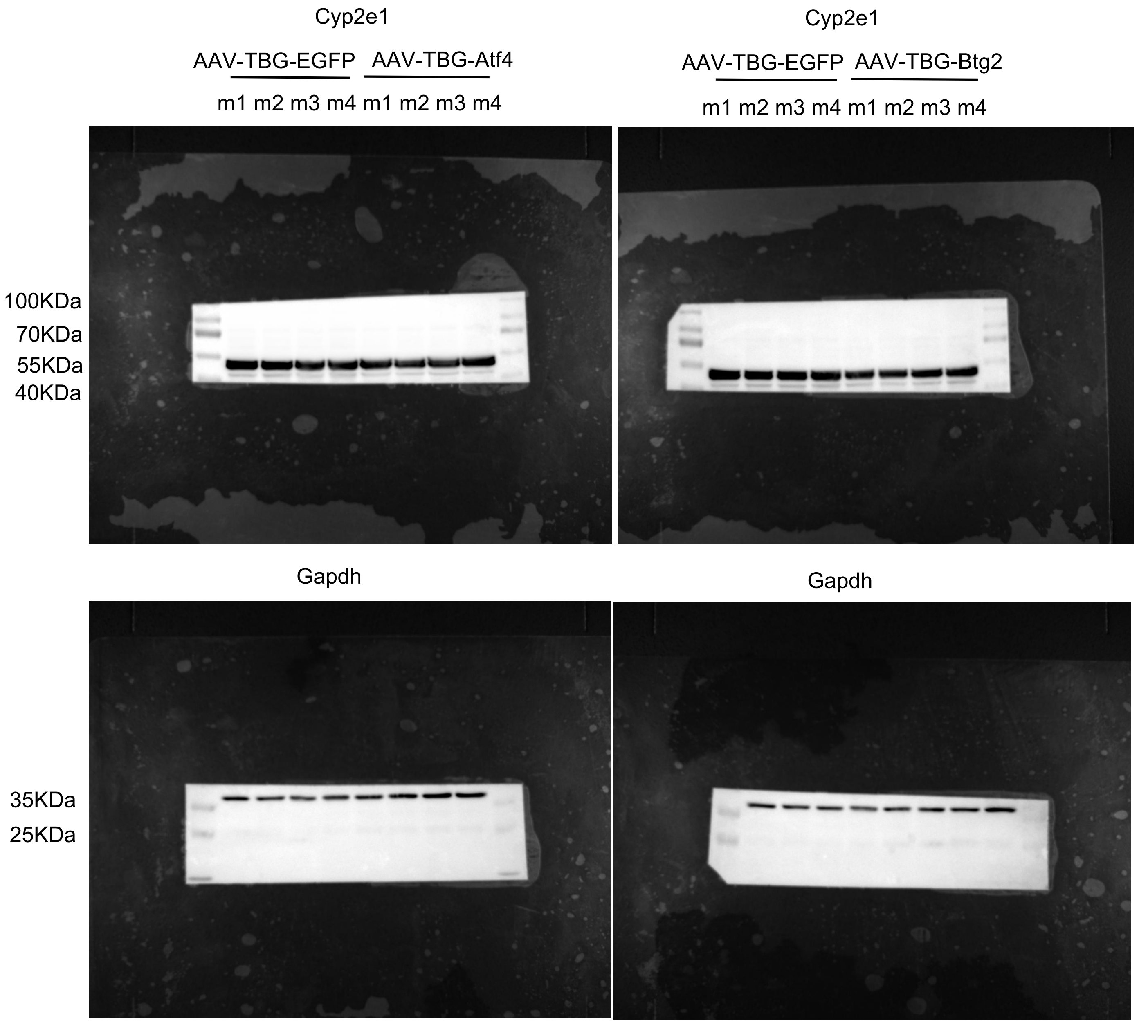

Figure 6-Figure supplement 1. Western blot analysis of Cyp2e1 protein in whole liver lysates from mice transduced with 1.2×10^{11} viral genome copies of AAV-TBG-EGFP, AAV-TBG-Atf4, or AAV-TBG-Btg2. Cyp2e1/Gapdh ratios are shown. n = 4 mice/group.
