# Supplementary material for: Mid-zone hepatocytes trade proliferation for survival via Atf4-Chop axis in early acute liver injury
Source: eLife. 2026 Oct 26;14:RP108646. doi: 10.7554/eLife.108646 (PMC13707374; doi:10.7554/eLife.108646)
Supplement: Supplementary file 3 [file elife-108646-supp1.docx]

**Supplementary file 1**

**qPCR primers**

| Targeted gene | Primer (5’-3’) | Sequence |
| --- | --- | --- |
| mus*Btg2* | Forward | ATGAGCCACGGGAAGAGAAC |
|  | Reverse | GCCCTACTGAAAACCTTGAGTC |
