# Supplementary material for: Mid-zone hepatocytes trade proliferation for survival via Atf4-Chop axis in early acute liver injury
Source: eLife. 2026 Oct 26;14:RP108646. doi: 10.7554/eLife.108646 (PMC13707374; doi:10.7554/eLife.108646)
Supplement: Peer review history

# Editor report: eLife Assessment

**DOI:** 10.7554/eLife.108646.4.sa0

**Authors:** Hao Zhu

**Affiliations:** The University of Texas Southwestern Medical Center United States

**Keywords:** Convincing, Solid, Important

The manuscript is **important** in that it has the potential to advance our understanding of how liver regeneration is orchestrated after injury of pericentral hepatocytes, which are a target of many drugs and toxins. The findings are **solid** and suggest that midlobular hepatocytes are protected from toxin-induced injury by a genetic program delaying proliferation to prevent cell death. The effects of individual actors in the proposed program are **convincingly** shown but whether they act in concert and whether cell cycle arrest is indeed the protective mechanism remains to be determined.

# Referee report: Reviewer #2 (Public review)

**DOI:** 10.7554/eLife.108646.4.sa1

The manuscript reports protection of midlobular hepatocytes from APAP toxicity by activation of Atf4-CHOP (Ddit3)-mediated cell cycle arrest and stress response. The authors acknowledge that their finding is unexpected because CHOP typically induces cell death. Therefore, they functionally validate several aspects of the proposed Atf4-CHOP mechanism. Along these lines, the mitigation of APAP toxicity by AAV expression of Atf4 or Btg2, the latter identified as CHOP effector, is impressive. Whether Atf4 indeed acts through CHOP and whether midlobular hepatocytes are protected because of cell cycle arrest is less clear. These and other criticisms are described in the following.

Major points:

(1) Starting with the basics, one wonders why midlobular hepatocytes manage to mount a defensive response to APAP but PC hepatocytes don't. Is this because midlobular hepatocytes express the relevant Cyps (2e1 but also 1a2 and 3a11) at lower levels, which mitigates toxicity and buys them time? This would be supported by F2A but not by F3B, at least not for the most important Cyp2e1. A moderate difference is shown for Cyp1a2 expression in F3D but is that enough to explain the different fates? Or are additional post-transcriptional effects on these Cyps at work? In the re-revised manuscript, it was clarified in the legends of F2A and F3B that they visualize the same data in different ways.

(2) The evidence presented in support of cell cycle arrest of midlobular hepatocytes is not fully convincing: there is no overt difference in S and G2/M gene scores in F2F; the marker genes used for S phase and G1 to S progression in F2G are unusual. Along these lines, one wonders if spatial transcriptomics confirmed the Ki67 immunostaining results in F1 also for specific zones, not only overall, as shown in F2E? The discussion and abstract of the re-revised manuscript acknowledge that spatial transcriptomics did not independently confirm cell cycle arrest in midlobular hepatocytes.

(3) The authors conclude in line 364 that halting of proliferation by Btg2 favors survival, which raises the question of whether Btg2 knockout causes death in midlobular hepatocytes in F6K. Data addressing this question, that is, localization and extent of tissue necrosis and ALT levels after APAP, are missing. The efficiency of knockout of Btg2 is also not given. Additional Btg2 knockout data support its proposed role in the revised manuscript.

(4) Related to the previous question, the BTG2 immunostaining in F6F is not convincing when compared to F6D. One also wonders if it is necessary to apply APAP to find induction of BTG2 by AAV-Ddit3? The text of the re-revised manuscript reflects that issues with immunostaining did not allow for concluding that APAP promotes nuclear localization of BTG2.

(5) Related to the previous question, the proposed Atf4-Ddit3 axis is challenged by the lack of midlobular induction of Atf4 in the APAP scRNA-seq data published by another group presented in S4F and G. Further analysis of AAV-Atf4 samples generated for F5 could address if it is really Atf4 that acts on Ddit3 in APAP toxicity. The extended list of transcription factors (from 30 to 50) includes Atf4 but direct evidence for an interaction with Ddit3 is missing from the revised manuscript. The re-revised manuscript acknowledges this limitation by referring to a Atf4-Chop (Ddit3) axis and defining that as a functional pathway, not direct interaction of the proteins.

(6) Related to the previous question, the ATF4 immunostaining in F5A doesn't look convincing, with many brown pigments appearing to be outside of the nucleus, which was addressed by adding high-magnification images to F5A of the re-revised manuscript.

(7) It is not ruled out that AAV expression of Atf4 or Btg2 reduces hepatocyte sensitivity to APAP by affecting expression of the Cyps needed for activation. In other words, does AAV-Atf4 or AAV-Btg2 change the expression of any of the Cyps relevant to APAP in the 3 weeks before APAP application (F5B)? S5A of the revised manuscript rules out loss of Cyp2e1 expression as a confounding factor.

(8) It is laudable that the authors tried to extend their findings to human by using snRNA-seq data from a published study (line 391) but it is unclear why they didn't analyze all 10 patients in that study but instead focused on 2 and stated that this small sample number prevented drawing definitive conclusions and could therefore only be mentioned in the discussion. The re-revised manuscript includes analysis of the more substantial snRNA-seq dataset (in addition to the limited spatial transcriptomics) of patients with APAP toxicity in S4-3E, which confirms the midlobular expression of stress-response genes observed in mice but differs from mice in activation of proliferation genes, which may be due to sampling at later stages of the injury response as explained in the text.

Minor points:

(1) What is the functional classification of DEG in F2A based on? GO terms? Clarified in revised manuscript.

(2) The rationale for focusing on CHOP is not clear because Ddit3 is not shown in the spatial transcriptomics in F2A and not significant in F2B, contradicting what is stated in line 206. F2A includes Ddit3 in the revised manuscript and although it is not significant in S1G (former F2B), it is among the most highly expressed transcription factors in F4B and S3B.

(3) The term "redistribution" used in line 197 to describe expression of Cyp2e1 and other Cyps in the midlobular zone seems inappropriate considering that they just continue to be expressed there whereas PC hepatocytes are dying in F3B; the same applies to "Gene Expression Shift" in F3H. Clarified in revised manuscript.

Comments on revised version.

The revised manuscript addressed many of the original points and the re-revised manuscript clearly describes remaining uncertainties, which may be technical in nature such as lack of cell cycle arrest of midlobular hepatocytes in spatial transcriptomics or could be addressed in follow-up studies such as the nature of the interaction between Atf4 and CHOP.

# Author comment: Author response

**DOI:** 10.7554/eLife.108646.4.sa2

**Authors:** Yaying Zhu, Chengxiang Deng, Bo Chen, Jia He, Yanan Liu, Shan Lei, Weiju Lu, Cheng Peng, Zhao Shan

**Affiliations:** Yunnan University Kunming China; Yunnan University kunming China; Yunnan University Kunming China; Yunnan University Kunming China; Yunnan University Kunming China; Yunnan University Kunming China; Yunnan University Kunming China; Yunnan University kunming China; Yunnan University Kunming China

The following is the authors’ response to the previous reviews.

**eLife Assessment**

This study addresses an important question in liver biology: how zonal hepatocytes balance survival and proliferation following injury? The authors propose that a midzone Atf4-Chop axis to Btg2 program temporarily suppresses proliferation to promote survival after a variety of chemical and surgical liver injury models. The authors provide evidence that some zones mount tailored stress responses, which ultimately promote regeneration and liver healing; however, the "mid-zone" changes with different injury models, making it difficult to conclude that the ATF4-CHOP response is specific to this zone in all injury contexts. In addition, it is possible that Atf4 and Btg2 overexpression could lead to Cyp2e1 suppression, which could reduce the extent of injury after CCl4 or APAP. To some extent, these points make the strength of the evidence incomplete, but do not entirely detract from the significance of the study, which is underscored by the helpful observation that there are zone-specific stress responses that mediate liver regeneration and survival.

We appreciate the editor’s thoughtful assessment. Regarding regional specificity, we agree that "mid-zone" patterns differ across injury models, particularly in HPx; to maintain consistency, we have removed the PHx data and now focus our conclusions on the APAP and CCl_4_ models. Concerning the potential confound of Cyp2e1 suppression, our AAV overexpression resulted in only modest reductions in Cyp2e1 protein (Revised Figure 4-figure supplement 3A). Although enzymatic activity was not directly measured, basal CYP2E1 protein levels generally correlate with enzymatic activity. Furthermore, published studies indicate that protection from APAP toxicity requires >50% CYP2E1 suppression (PMID: 35145060; PMID: 30151903), whereas the reduction observed here was substantially smaller and therefore unlikely to account for the marked decrease in liver injury.

**Reviewer #2 (Public review):**

The manuscript reports protection of midlobular hepatocytes from APAP toxicity by activation of Atf4-CHOP (Ddit3)-mediated cell cycle arrest and stress response. The authors acknowledge that their finding is unexpected because CHOP typically induces cell death. Therefore, they functionally validate several aspects of the proposed Atf4-CHOP mechanism. Along these lines, the mitigation of APAP toxicity by AAV expression of Atf4 or Btg2, the latter identified as CHOP effector, is impressive. Whether Atf4 indeed acts through CHOP and whether midlobular hepatocytes are protected because of cell cycle arrest is less clear. These and other criticisms are described in the following.

Major points:

(1) The difference in baseline Cyp2e1 expression between F2A and F3B remains unexplained after revision.

We apologize for the confusion. The apparent discrepancy reflects different visualization methods rather than a biological inconsistency. Figure 2A and Figure 3B are derived from the same spatial transcriptomic dataset but are visualized using different normalization strategies. Figure 2A uses row-wise Z-score normalization to emphasize the relative spatial distribution of each gene across liver zones. Consequently, even a moderate spatial gradient (e.g., ~1.5–2-fold) is visually enhanced to facilitate comparison of zonation patterns between genes. In contrast, Figure 3B presents the absolute log-normalized transcript abundance without rowwise scaling. Because Cyp2e1 is highly expressed in both the pericentral and adjacent mid-zone hepatocytes, the absolute difference between these regions is relatively modest, resulting in a less pronounced visual contrast. Thus, the different appearance of Cyp2e1 between Figures 2A and 3B reflects the visualization strategy rather than conflicting expression data. To avoid further confusion, we have added this clarification to the legends of both Figures 2A and 3B.

(2) In contrast to the revised discussion, the abstract does not reflect that limited evidence for a cell cycle arrest in pericentral hepatocytes was found.

We thank the reviewer for the continued effort in improving our manuscript. To reflect the limited support of ST data for cell cycle arrest, we have revised the Abstract (Revised manuscript, page 1, line 13-26)

(3) Additional Btg2 knockout data support its proposed role in the revised manuscript.

We appreciate the reviewer’s acknowledgement of the new functional data supporting the role of Btg2.

(4) The BTG2 immunostaining remains weak, not only in in F6F but now also in F6D of the revised manuscript, which together with lack of high-resolution immunostaining of AAV-Ddit3-induced BTG2 in the absence of APAP results in limited support for the conclusion that APAP promotes nuclear localization of BTG2.

We agree that the current immunostaining does not provide definitive evidence for nuclear localization. In response, we have deliberately avoided any claims regarding nuclear translocation in the revised manuscript, instead framing our findings strictly around increased BTG2 expression and accumulation in the peri-necrotic mid-zone region following APAP injury.

(5) The extended list of transcription factors (from 30 to 50) includes Atf4 but direct evidence for an interaction with Ddit3 is missing from the revised manuscript.

We thank the reviewer for this important point. We agree that our current dataset does not provide direct evidence for a physical interaction between Atf4 and Ddit3 proteins. Throughout the manuscript, we use the term "Atf4–Chop axis" to denote a functional regulatory pathway rather than a direct protein–protein interaction. To avoid ambiguity, we have revised the manuscript to characterize Atf4 and Ddit3 as co-activated transcriptional co-regulators and to restrict our conclusions to transcriptional co-regulation and pathway convergence, as supported by SCENIC, Cut&Run, and GO analyses. We now explicitly define "axis" as a functional pathway (revised manuscript, page 5, line 182–184; page 6, line 260–261; page 8, line 331333).

(6) The ATF4 immunostaining after APAP challenge remains weak.

We agree that the endogenous ATF4 immunostaining is relatively weak. This is consistent with the low basal abundance and transient induction of ATF4 protein during the integrated stress response. To improve visualization, we have added higher-magnification insets to Revised Figure 5A, which more clearly show nuclear ATF4 staining in hepatocytes adjacent to the necrotic region following APAP treatment. We hope these enlarged images better illustrate the spatial distribution of ATF4 while accurately reflecting its endogenous expression level.

(7) S5A of the revised manuscript rules out loss of Cyp2e1 expression as a confounding factor.

We thank the reviewer for acknowledging that Revised Figure S5A addresses this concern.

(8) The revised manuscript continues to focus on rare spatial transcriptomics analyses of patients with APAP toxicity although more snRNA-seq analyses of such patients are available which should also allow for analysis of hepatocyte zonation.

We thank the reviewer for this constructive suggestion and apologize for not describing our analyses more clearly in the previous revision. In response, we analyzed both the snRNA-seq and spatial transcriptomics (ST) datasets from GSE223561, which includes snRNA-seq profiles from 9 healthy and 10 APAP explants together with ST data from 3 healthy and 2 APAP patients (Revised Figure 4-figure supplement 3).

The snRNA-seq data readily resolved hepatocyte zonation using established pericentral (Glul, Cyp2e1, Cyp2a5), periportal (Alb, Cyp2f2, Sds), and mid-zonal (Igfbp2, Hamp) markers (Figure 1A–D). Consistent with our mouse data, mid-zonal hepatocytes in APAP patients showed increased expression of stress-response genes (DDIT3, ATF4, and HMOX1). However, unlike the early mouse injury model, proliferation-associated genes (MKI67 and CCNB1) were also elevated (Revised Figure 4-figure supplement 3E), likely reflecting the end-stage nature of liver explants, in which regenerative responses are already well established rather than the early (3–12 h) injury phase examined in our study.

The ST dataset provided complementary spatial information that cannot be obtained from dissociated nuclei alone. Although limited in sample number, one APAP specimen closely recapitulated our murine observations, showing mid-zonal stress enrichment accompanied by reduced proliferation, whereas the second exhibited a predominantly proliferative profile consistent with a later regenerative stage (Revised Figure 4-figure supplement 3F).

Together, these analyses indicate that human mid-zonal hepatocytes exhibit a conserved stress-response program, while the relationship between stress signaling and proliferation is highly dependent on the stage of injury. We have incorporated both the snRNA-seq and ST analyses into the revised manuscript and explicitly discuss the temporal limitations of the available human datasets (page 5-6, lines 225239; page 9, lines 368-379).

**Recommendations for the authors:**

**Reviewer #2 (Recommendations for the authors):**

(1) F2A includes Ddit3 in the revised manuscript and although it is not significant in S1G (former F2B), it is among the most highly expressed transcription factors in F4B and S3B.

We thank the reviewer for this careful observation. As noted, Ddit3 is differentially expressed in the mid-zonal hepatocytes at both 3 and 6 h after APAP treatment (Revised Figure 2A). We also agree that Ddit3 does not reach statistical significance in the differential expression analysis shown in Revised Figure 2-figure supplement 1G under our predefined thresholds. However, our rationale for prioritizing Ddit3 was based primarily on transcription factor activity rather than differential expression alone. Differential expression analysis evaluates changes in the abundance of an individual transcript, whereas SCENIC regulon analysis infers the functional activity of a transcription factor from the coordinated expression of its downstream target genes. Consequently, transcription factors with relatively modest changes in mRNA abundance may nevertheless exhibit high regulon activity if their downstream regulatory network is broadly activated.

Consistent with this, Ddit3 was identified as the highest-activity regulon in the mid zone, while Atf4 ranked seventh (Figures 4B and Figure 4-figure supplement 1B). Given the well-established cooperative roles of ATF4 and DDIT3 in the integrated stress response, these unbiased network analyses identified the ATF4–DDIT3 axis as a biologically relevant pathway for further investigation. To avoid overinterpretation, we have revised the manuscript to clarify that DDIT3 was prioritized based on its consistently high regulon activity and the activation of its downstream transcriptional network, rather than on differential expression alone (Revised manuscript, page 5, line 188-192).

We thank the reviewers for their rigorous critique again. We thank eLife for fostering an environment of fairness and transparency that enables authors to communicate openly and present their data honestly.
